# Supplementary material for: A detachable interface for stable low-voltage stretchable transistor arrays and high-resolution X-ray imaging
Source: Nat Commun. 2024 Mar 23;15:2624. doi: 10.1038/s41467-024-47026-9 (PMC10960804; doi:10.1038/s41467-024-47026-9)
Supplement: Supplementary file 1 — Supplementary information [file 41467_2024_47026_MOESM1_ESM.pdf]

SUPPLEMENTARY INFORMATION

A detachable interface for stable low-voltage stretchable transistor arrays and high-resolution X-ray imaging

Yangshuang Bian, Mingliang Zhu, Chengyu Wang, Kai Liu, Wenkang Shi, Zhiheng Zhu, Mingcong Qin, Fan Zhang, Zhiyuan Zhao, Hanlin Wang, Yunqi Liu and Yunlong Guo*

*Corresponding author. Email: guoyunlong@iccas.ac.cn (Y.G.)

Section 1. Preparation and characterization of micropatterned electrodes assisted by the detachable interface for high-density intrinsically stretchable organic transistor arrays:

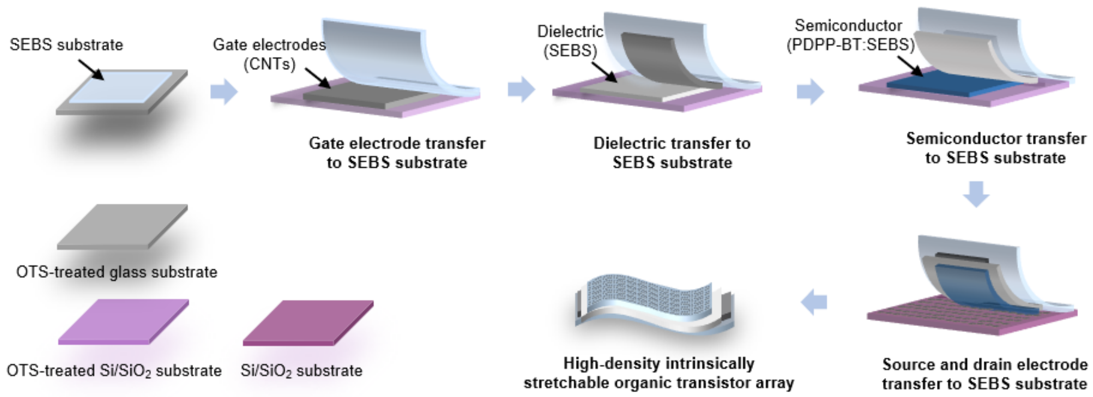

Supplementary Figure 1. Diagram illustration of the fabrication process of high-density intrinsically stretchable organic transistor array. The thickness of SEBS substrate can be altered from ~10 μm (spin-coating method) to ~1 mm (drop-casting method) depending on the practical requirements. The thickness of the electrode is 30~40 nm (spray-coating method). The thickness of the dielectric layer and semiconductor layer are about 1200 nm and 100 nm, respectively. The substrates of gate electrodes, dielectric and semiconductor employed OTS-treated Si/SiO_2 wafer. The source and drain electrodes was fabricated on a cleaned Si/SiO_2 wafer by the left-off technology with the assistance of a LiF layer, as shown in Fig.1a.

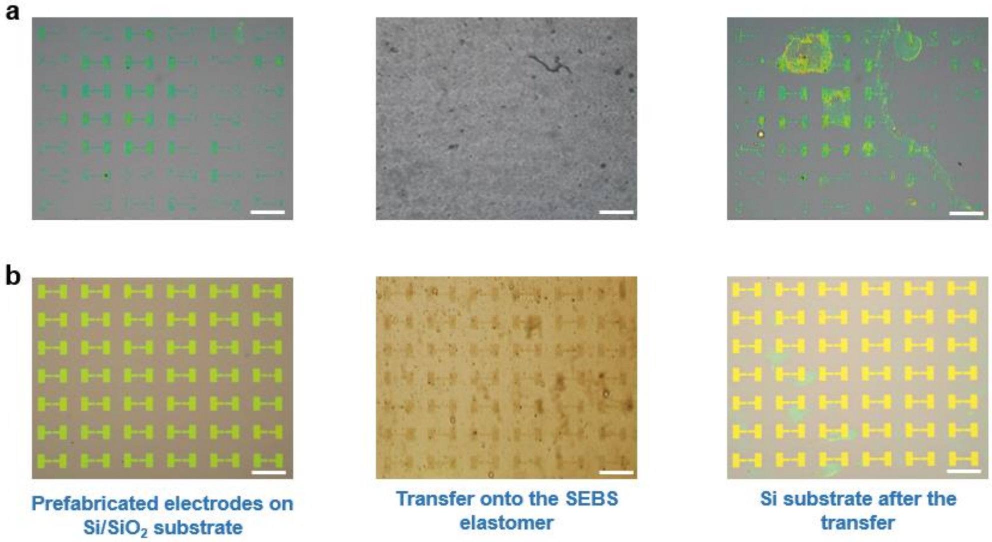

Supplementary Figure 2. Optical microscope images of the micropatterned stretchable CNT electrodes during the prefabrication and transfer process. a, Direct lift-off technology for the integration of intrinsically stretchable CNT electrodes. **b,** LiF-assisted lift-off technology for the integration of intrinsically stretchable CNT electrodes (The unclean surface was caused by the transparent SEBS substrates, which had no obvious influence on the transfer of CNT electrodes). The scan bar is 100 µm.

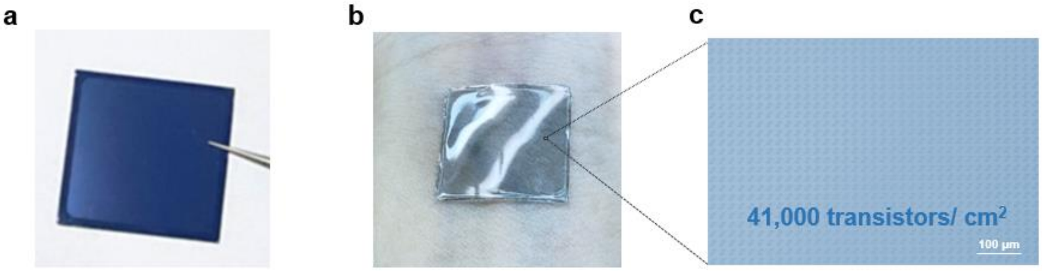

Supplementary Figure 3. High-resolution stretchable CNT electrodes for high-density intrinsically stretchable organic transistor arrays. a, Photograph of the pre-fabricated high-resolution stretchable CNT electrodes with a detachable interface over a 2 cm ×2 cm substrate. **b,** Photograph of the high-density intrinsically stretchable organic transistor array. **c,** Optical microscope image showing the integrated intrinsically stretchable organic transistor matrix with a high density of 41,000 /cm^2.

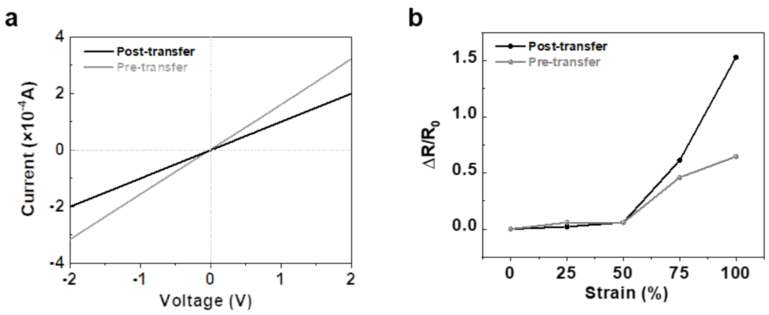

Supplementary Figure 4. Electrical characteristics of the micropatterned CNT electrodes after transfer onto the stretchable substrate with the assistance of a detachable interface. a, Conductivity of the high-resolution CNT electrodes before and after the transfer (The slightly decreased conductivity might be caused by the insulative SEBS substrate). **b,** Normalized resistance change as a function of strain for the high-resolution stretchable CNT electrodes before and after the transfer assisted by a detachable interface.

Calculation of the surface energies of the CNTs and LiF/CNTs used in this study:

The water and diiodomethane contact angle of polymer semiconductor films were measured by the Drop Shape Analyzer (DSA100, KRÜSS) in static mode at room temperature. The obtained contact angle from the average of the left and right angles of a sessile drop was measured by KRÜSS software based on the tangential method. The de-ionized water and diiodomethane (≈1 μL) were dropped on the CNTs and LiF/CNTs, and then the droplet was snapshotted after the equilibrium on the gas-liquid-solid interface. The contact angle need to be fixed at the standard deviation of ±0.5°.[1, 2] The surface energies of CNTs and LiF/CNTs were calculated by the Owens-Wendt method equation[3]:

$$\gamma_s = \gamma_s^p + \gamma_s^d$$

$$(1 + cos\theta_l)\gamma_l = 2(\sqrt{\gamma_l^d \gamma_s^d} + \sqrt{\gamma_l^p \gamma_s^p})$$

Where θ_l represents the contact angle of the testing liquid, including de-ionized water and diiodomethane. Thereinto,γ_s, γ_s^p and γ_s^d are the total surface energy, polar component and dispersion component of the sample, respectively. The total surface energy (γ_l), dispersion component (γ_l^d)and polar component (γ_l^p) are 50.8 mJ m^{-2}, 50.42 mJ m^{-2} and 0.38 mJ m^{-2} for diiodomethane, and 72.8 mJ m^{-2}, 29.1 mJ m^{-2} and 43.7 mJ m^{-2} for water, respectively.

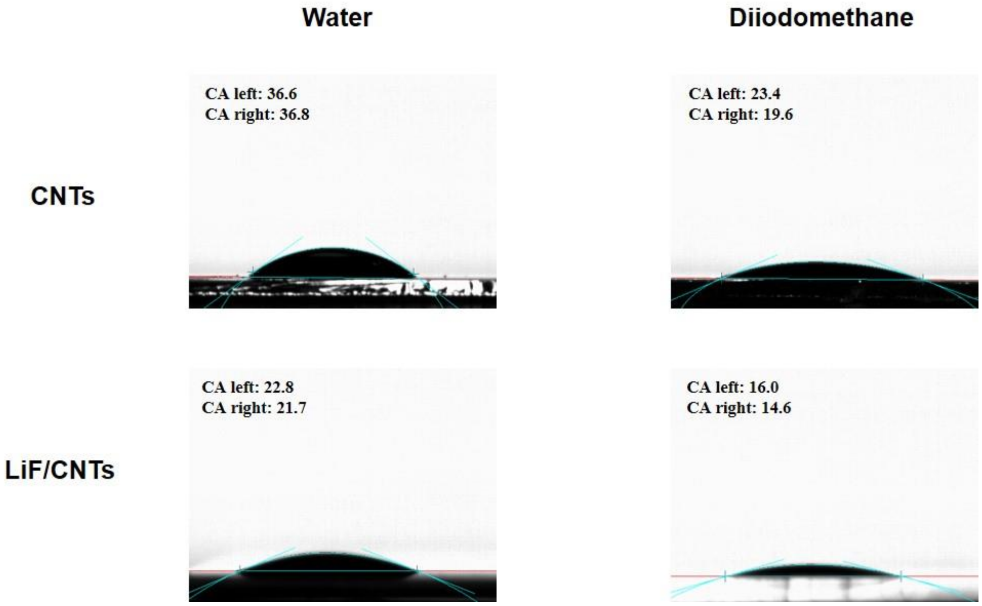

Supplementary Figure 5. Optical images of the snapshotted droplet showing the contact angels of the CNTs and LiF/CNTs.

Supplementary Table 1. Surface energies of the CNTs and LiF/CNTs.

**Materials**	**Contact angle (°)**		**Surface energy (mJ/m^2)**		
	CH_2I_2	H_2O	γ_s^d	γ_s^p	γ_s
CNTs	21.5	36.7	42.25	21.2	63.45
**LiF/CNTs**	**15.3**	**22.2**	**43.13**	**27.27**	**70.4**

Section 2. Electrical performance of the high-density intrinsically stretchable organic transistor arrays:

The mobility (μ), threshold voltage (V_{th}) and subthreshold swing (SS) are important parameters for evaluating the electrical performances of OFETs. These values can be extracted from the curve of the drain current (I_{DS}) versus the gate voltage (V_G). In the saturation regime, the standard equation is[4]:

$$I_{DS} = \frac{W}{2L} C_i \mu_{sat} \left(V_G - V_{th}\right)^2$$

Where W and L represent the channel width and length, respectively. C_i is the capacitance of the dielectric layer.

In details, the mobility is calculated by the slop of $\sqrt{|I_{DS}|}$ versus V_G according to the following equation[5]:

$$\mu_{sat} = \left(\frac{\partial\sqrt{|I_{DS}|}}{\partial V_G}\right)^2 \frac{2L}{WCi}, \text{ applicable at } |V_G - V_{th}| < |V_{DS}|$$

The V_{th} of the device is obtained by extrapolating a plot of $\sqrt{|I_{DS}|}$ versus V_G to $I_{DS} = 0$.

The subthreshold swing is defined as[4]:

$$SS = \mathrm{d}\, V_G / \mathrm{d}\,(\log I_{DS})$$

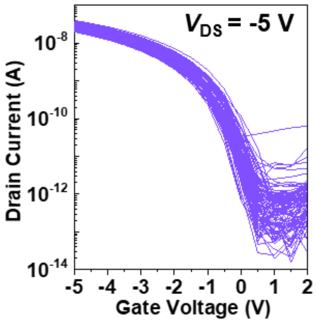

Supplementary Figure 6. Transfer curves (V_{DS} = -5 V) obtained from the 100 devices in a 10×10 stretchable organic transistor array with a channel length of 2 µm.

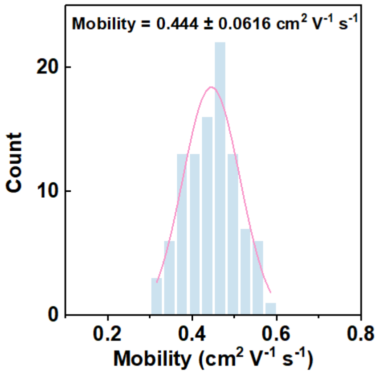

Supplementary Figure 7. Statistical distribution of mobility representing the device-to-device variation in a 10×10 stretchable organic transistor array with a channel length of 2 µm.

Supplementary Table 2. Device geometry and dielectric capacitance[3] (*d*=1200 nm) for the short-channel intrinsically stretchable organic transistor under different strains.

Stretching direction	Strain(%)	Channel length(μm)	Channel width(μm)	Capacitance (nF/cm^2)
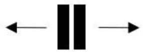 Channel length	0	2	8	1.5
	25	2.5	7.44	1.61
	50	3	6.88	1.74
	75	3.5	6.32	1.9
	100	4	5.76	2.08
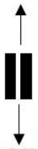 Channel width	0	2	8	1.5
	25	1.86	10	1.61
	50	1.72	12	1.74
	75	1.58	14	1.9
	100	1.44	16	2.08

Supplementary Table 3. Performance comparison of our short-channel intrinsically stretchable organic transistor with previously reported stretchable organic transistors based on CNT electrodes.

Source/drain electrodes	Semiconductor	Channel length (μm)	Operation voltage (V)	Subthreshold swing (V/dec)	Device density (transistors/cm^2)	Ref.
CNTs	DPP-TT/SEBS	200	40	5.5	*	3
CNTs	DPP-SVS/SEBS	200	15	2.5	*	5
CNTs	DPP-polymer	150	40	5	*	6
CNTs	DPPTT	150	28	7	*	7
CNTs	PIDTBT	150	20	4.5	*	8
CNTs	PDPPFT4	100	30	4.5	*	9
CNTs	TTA-DPP	50	25	8	*	10
CNTs	PSe-DPP	50	60	7.5	*	11
CNTs	P3HT	50	100	8	*	12
CNTs	DPP-TT/SEBS	200	5	0.5	8	13
CNTs	DPP-TT/SEBS	200	60	1.2	8	14
CNTs	FIID-CF_3TVT/SEBS	200	60	3	8	15
CNTs	DPP-SVS/SEBS	80	25	2.7	340	16
CNTs	DPP-SVS/SEBS	70	22	3	347	17
CNTs	PDPP-BT/SEBS	2	5	0.315	41000	This work

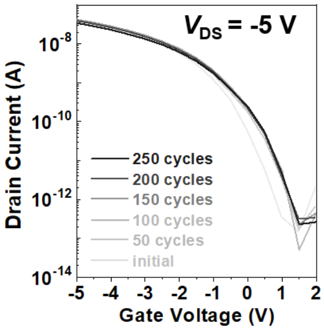

Supplementary Figure 8. Cyclic performance of the short-channel intrinsically stretchable organic transistor during the 250 consecutive scan test at V_{DS} = -5V.

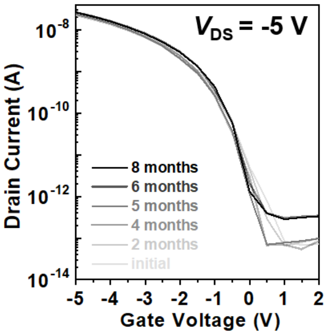

Supplementary Figure 9. Long-term stability of the short-channel intrinsically stretchable organic transistor stored for 8 months at V_{DS} = -5V.

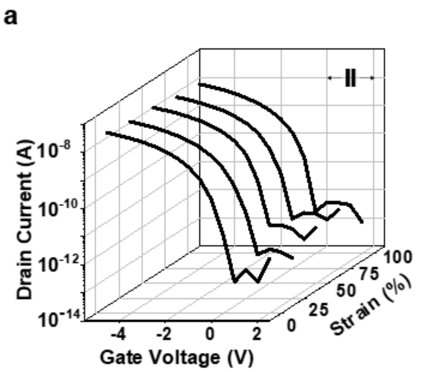

Supplementary Figure 10. Transfer curves (V_{DS} = -5 V) of the short-channel intrinsically stretchable organic transistor under different stretching strains. a, Transfer curves of the short-channel intrinsically stretchable organic transistor at different stretching strains parallel to the charge transport direction. **b,** Transfer curves of the device at different stretching strains perpendicular to the charge transport direction.

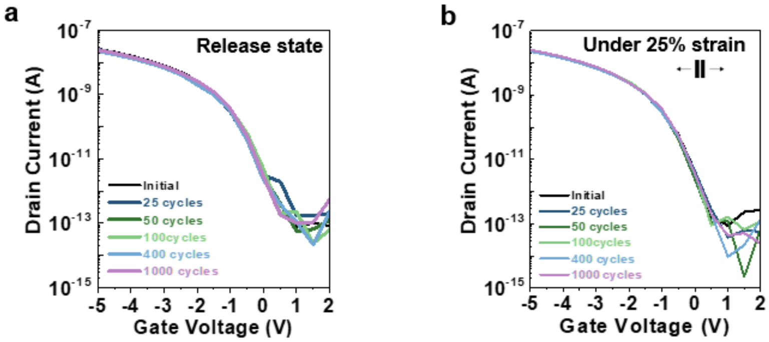

Supplementary Figure 11. Transfer curves (V_{DS} = -5V) of the short-channel intrinsically stretchable organic transistor subjected to multiple stretch-release cycles. **a,** Transfer curves of the short-channel intrinsically stretchable organic transistor after multiple stretching cycles with 25% strain parallel to the charge transport direction at the released state. **b,** Transfer curves of the device under multiple stretching cycles at 25% strain.

Section 3. Photosensitivity evaluation of the short-channel intrinsically stretchable organic transistor for X-ray detection.

1. Calculation of sensitivity

The sensitivity including the sensitivity per unit area (S_A) and sensitivity per unit volume(S_V), are defined as[18]:

$$S_A = \frac{I_{light} - I_{dark}}{DA}$$

where I_{light} and I_{dark} represent the photocurrent and dark current, respectively. D is the dose rate of X-ray irradiation, and A represents the active area of the organic semiconductor exposed under the X-ray irradiation.

Typically, the detection sensitivity can be derived from the slope of the fitting linear of the current density versus different dose rates. The expression is as follows[6]:

$$S_A = \partial J / \partial D$$

where J is the current density and d is the thickness of active layer. The current density is defined as[19-21]:

$$J = \frac{I}{WL}$$

where I is the current. W and L represent the width and length of the channel, respectively.

2. Calculation of signal-noise ratio

The expression of signal-noise ratio (SNR) is depicted as follows[22]:

$$SNR_{out} = \frac{\overline{X_\iota}}{\sigma_{X_i}}$$

where $\overline{X_\iota}$ and σ_{X_i} are the signal current and noise current, respectively. The signal current is defined as the average value of photocurrent (X_i):

$$\overline{X_\iota} = \langle X_i \rangle = \frac{1}{N}\sum_{i=1}^{N} X_i = \frac{1}{N}\sum_{i=1}^{N}\left(x_n^{i'} - \overline{x_{dark}}\right)$$

Where $\overline{x_{dark}}$ and x_n^i are the average value of dark current and data point of signal current, respective.

As for the noise current is determined by the standard deviation of signal current:

$$\sigma_{X_i}^2 = \langle (X_i - \overline{X_\iota}) \rangle = \frac{1}{N}\sum_{i=1}^{N}(X_i - \overline{X_\iota})^2$$

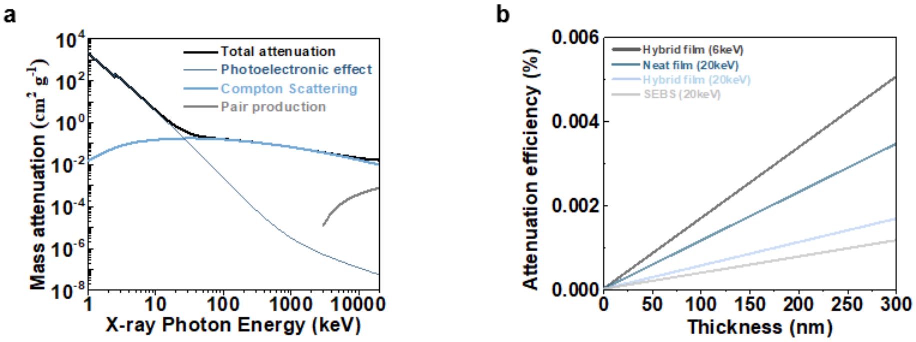

Supplementary Figure 12. Theoretical evaluation of the X-ray absorption of the photoactive materials. a, Total mass attenuation of X-rays by hybrid polymer semiconductor (PDPP-BT:SEBS) showing the contribution from photoelectric effect, compton scattering and pair production. **b,** Attenuation efficiency as a function of the film thickness for neat SEBS, PDPP-BT polymer and hybrid semiconductor films to 6 keV and 20 keV X-ray photons.

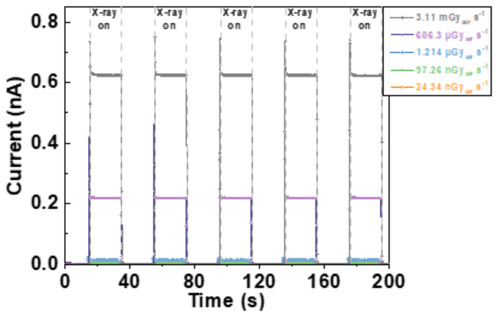

Supplementary Figure 13. Temporal response of the short-channel intrinsically stretchable organic transistor under different dose rates of X-ray irradiation at V_{DS} = -5V (V_G = 0.5 V).

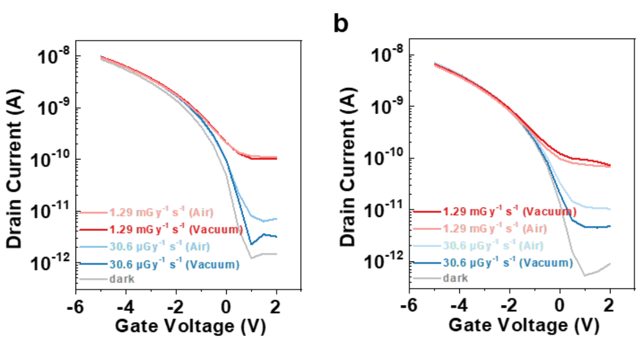

Supplementary Figure 14. Photoresponse comparison of stretchable short-channel organic transistors under X-ray irradiation in vacuum and air conditions. Transfer curves of stretchable short-channel organic transistors with two different bitches (**a,** bitch 1 and **b,** bitch 2) under X-ray irradiation in vacuum and air conditions.

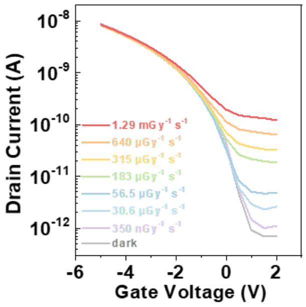

Supplementary Figure 15. Photoresponse curves of stretchable short-channel organic transistors under different dose rates in a vacuum (<100 Pa).

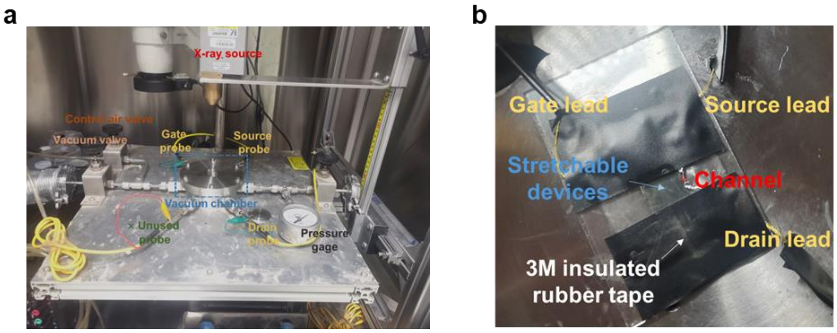

Supplementary Figure 16. Schematic diagrams of the vacuum measurement equipment. a, Photograph of the whole vacuum measurement equipment. **b,** Schematic of the connected stretchable short-channel organic transistor, in which the device and wires were not fully shielded for intuitive description.

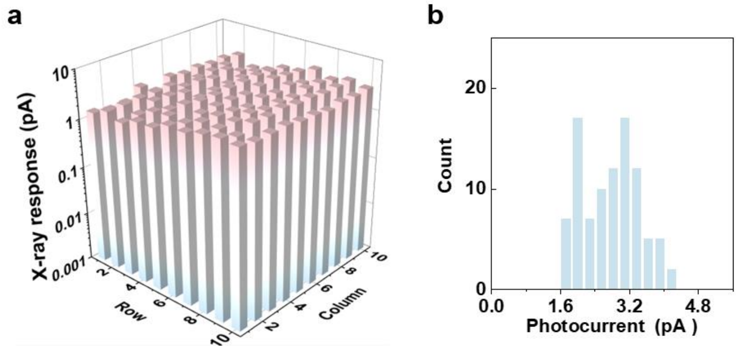

Supplementary Figure 17. Device-to-device variation of X-ray photocurrent in our short-channel stretchable transistor-based detectors. a, Histograms of X-ray induced photocurrent in a 10×10 organic transistor array at a low dose rate, down to 24.34 nGy_{air} s^{-1}. **b,** Statistical distribution of X-ray induced photocurrent calculated from the dynamic response of 100 devices measured at a low dose rate of 24.34 nGy_{air} s^{-1}, biased at V_{DS} = -5V and V_G = 0.5V.

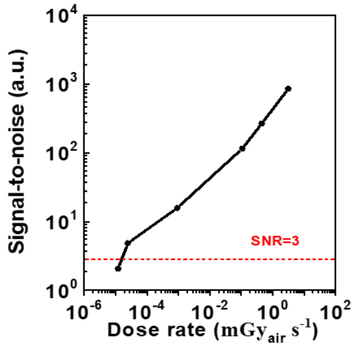

Supplementary Figure 18. Signal-to-noise ratio of the short-channel stretchable transistor-based detector derived by calculating the standard deviation of the X-ray signal current under different dose rates at V_{DS} = -5V (V_G = 0.5 V). The red dashed line represents a SNR of 3.

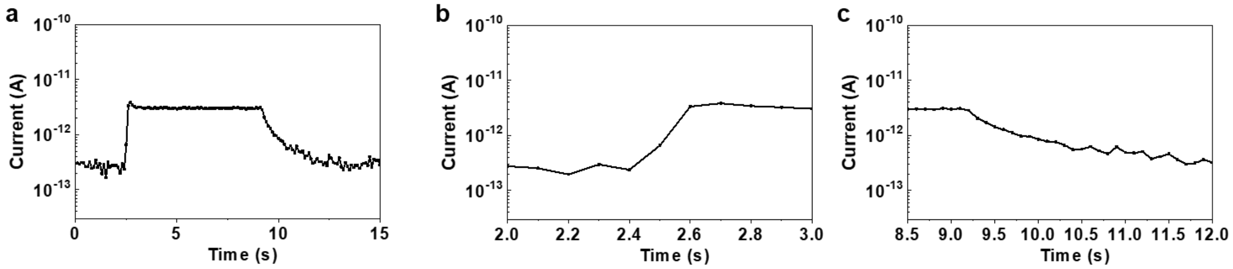

Supplementary Figure 19. High-resolution temporal response of the short-channel stretchable transistor-based detector under 24.34 nGy$_{air}$ s^{-1} dose rate at V_{DS} = -5V (V_G = 0.5 V). a, X-ray response of the short-channel stretchable transistor-based detector at 24.34 nGy$_{air}$ s^{-1} dose rate. **b,** High-resolution curve showing the rise time of the short-channel stretchable transistor-based detector at 24.34 nGy$_{air}$ s^{-1} dose rate. **c,** High-resolution curve showing the fall time of the short-channel stretchable transistor-based detector at 24.34 nGy$_{air}$ s^{-1} dose rate.

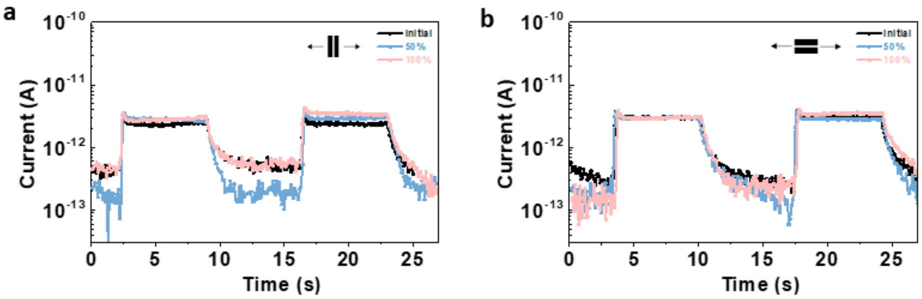

Supplementary Figure 20. Stretchable stability of the short-channel intrinsically stretchable organic transistor for X-ray detection. a, X-ray response of the intrinsically stretchable organic transistor with different strains parallel to the charge transport direction. **b,** X-ray response of the device with different strains perpendicular to the charge transport direction under 24.34 nGy$_{air}$ s^{-1} dose rate at V_{DS} = -5V (V_G = 0.5 V).

Supplementary Table S4. Comparison in the performance of the prepared short-channel intrinsically stretchable organic transistor with the previously reported X-ray detectors.

The type of devices	Photoactive materials	Structure	Operate Voltage (V)	Electric Field (V/μm)	X-ray source (anode voltage/ X-ray energy)	Minimum dose rate (mGy_{air} s^{-1})	S_A (μC Gy^{-1} cm^{-2})	Ref.
Perovskite detectors	$MAPbBr_{3-x}Cl_x$	Diode	-5	2.5×10^{-3}*	--; 8keV	7.60×10^{-6}	8.4×10^{4}	23
	$CsPbBr_3$	Diode	1.2	5×10^{-3}*	W; 50keV	3×10^{-2}	5.57×10^{4}	24
	$MAPbBr_3$	Diode	-1	5×10^{-4}*	Cu; --	3.6×10^{-2}	2.1×10^{4}	25
	$MAPbI_3$	Diode	30	0.03	W; 50kV	2.2×10^{-7}	1060	26
	$MAPbI_3$	Resistance	10	0.333*	Cu; 8keV	0.928	650*	27
	$(NH_4)_3Bi_2I_9$	Resistance	10	6.5×10^{-3}*	Ag;50keV	5.50×10^{-5}	803	28
	$Cs_2AgBiBr_6$	Resistance	50	0.025*	W; 50kV	5.2×10^{-3}	105	29
Direct organic inorganic hybrid X-ray detectors	B_2O_3/P3HT:PCBM	Diode	-10	0.5*	W; 50kV	0.13	4.79*	30
	Ta/F8T2	Diode	-50	2.5*	Mo;17.5keV	5	0.217*	31
	B_2O_3/PTAA	Diode	-100	20*	Mo;17.5keV	13	0.15*	
	PbS/P3HT:PCBM	Diode	-30	0.067*	W; 40kV	10	3.66*	32
	$CsPbBrI_2$/DPP-TT	Transistor	-60	1.5*	--; 20 kV	1.0×10^{-3}	3×10^{9}	20
Indirect organic inorganic hybrid X-ray detectors	GOS:Tb/P3HT:PCBM	Diode	-10	10	W; 70kV	1.5	7.35*	33
	$CsPbBr_3$/P3HT:PCBM	Diode	-3	0.2	Cu; 40–80 kV	58.18	3.67	34
Direct organic X-ray detectors	PFO	Diode	-50	2.5*	Mo; 50keV	4	0.96*	35
	MEH-PPV	Diode	-10	0.5*	Mo; 50keV	4	0.48*	
	P3HT	Diode	44	1.517*	--	16.6	0.47*	36
	TIPS-pentacene	Resistance	0.1	0.057*	Mo;17 keV	55	0.77*	37
	TIPGe-pentacene	Transistor	-3	0.067*	Mo; 35 kV	6.4×10^{-3}	18*	19
	TIPS-pentacene:PS	Transistor	-20	0.8*	Mo; 35 kV	3.5×10^{-2}	1.3×10^{4}	21
	FIID-CF_3TVT:SEBS	Transistor	60	0.3*	Ag; 20 keV	3.77×10^{-5}	1.52×10^{4}	15
	PDPP-BT/SEBS	Transistor	-5	2.5	Ag; 7 kV	2.4×10^{-5}	5.74×10^{6}	This work

*Values extracted by the device information reported in the referenced papers.

Section 4. X-ray imaging of the intrinsically stretchable organic transistor-based image sensors.

Resolution is the level of detail in distinguishing objects that can be expressed in line-pairs per millimeter (which is known as frequency, lp mm^{-1}). The line-pair represents a sequence of alternated black and white line. Bar target measurement is an ideal mean for the evaluation of system resolution, using direct image of the white and black bars with different frequencies. Modulation transfer function (MTF) is an important parameter to calculate imaging capability of the image sensor at different spatial frequencies. The MTF value is often extracted from the max and min brightness in the image of a bar-pair target according to the following equation[38]:

$$\mathrm{MTF} = \frac{I_{\mathrm{max}} - I_{\mathrm{min}}}{I_{\mathrm{max}} + I_{\mathrm{min}}}$$

Where I_{max} and I_{min} represent the max and min current extracted from the white and black line in the resultant image, respectively.

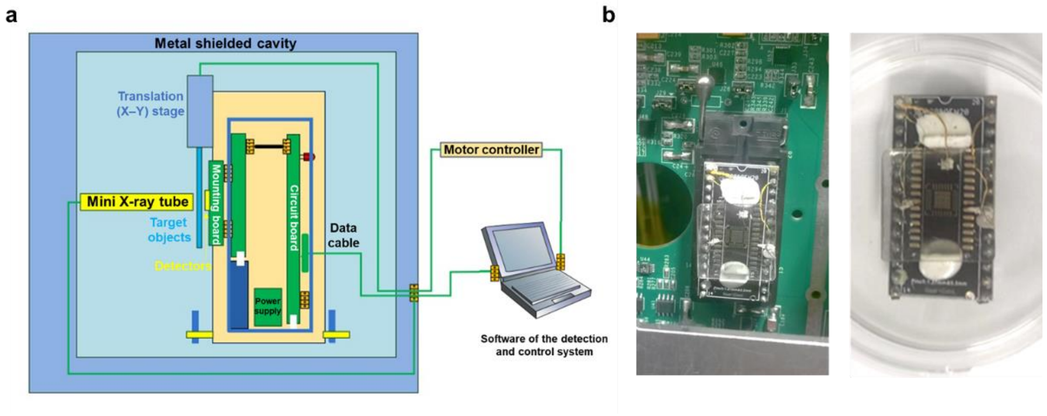

Supplementary Figure 21. Schematic illustration of the single-pixel imaging system. a, Schematic of the single-pixel imaging equipment. **b,** Photograph of the practical mounting boards connected with our stretchable transistors.

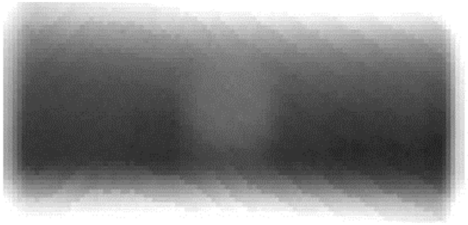

Supplementary Figure 22. X-ray image of the steel sheet with a round hole using the large-size stretchable organic transistor (*W*/*L*=4000 µm/200 µm) fabricated by previous reported method[3] (The scanning spacing is 400 µm).

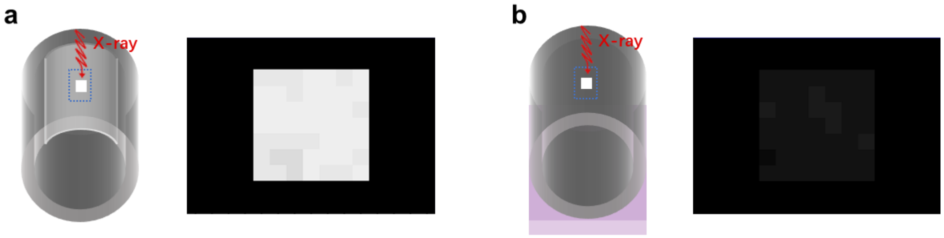

Supplementary Figure 23. Schematic and experimental results of the hole of the pipe in the wall imaged by the stretchable detectors from inside (a) and the rigid detectors from outside (b) (All images were taken without noise subtraction).

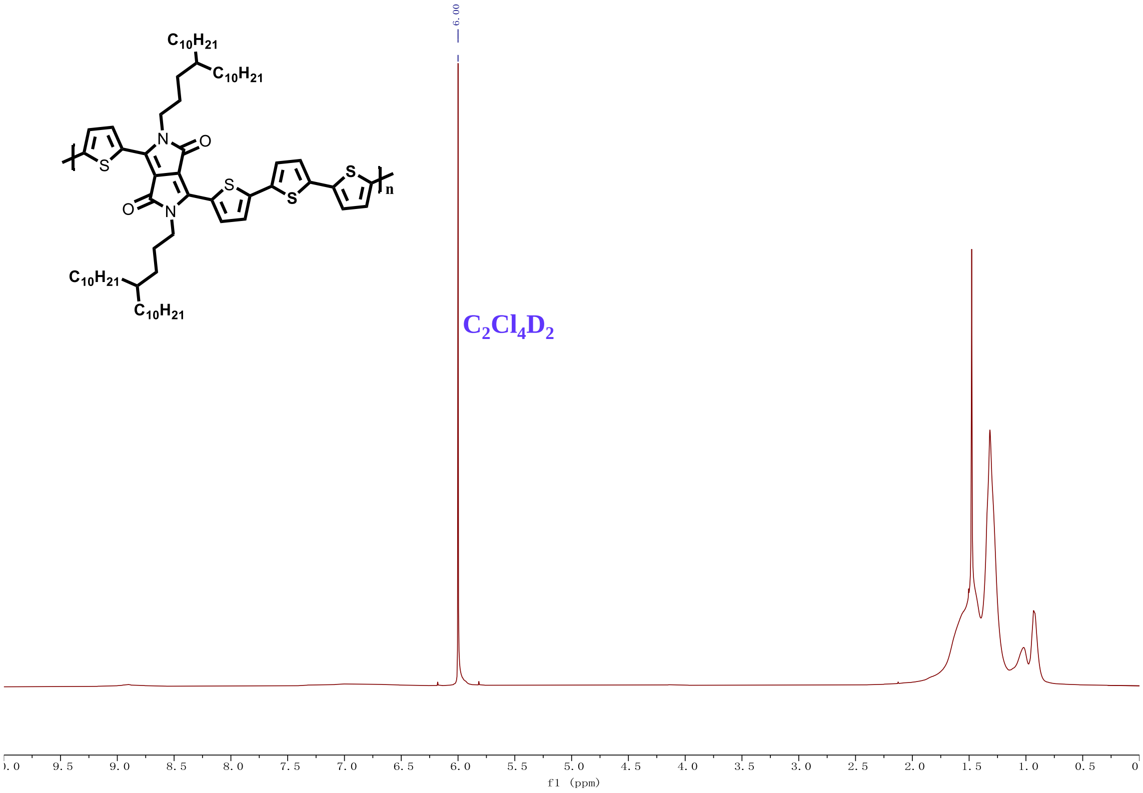

Supplementary Figure 24. H NMR spectrum of PDPP-BT in 1,1,2,2-tetrachloroethane-d_2 at 100°C.

References

Reference

1. Caddeo, C. et al. Hydrophilicity and water contact angle on methylammonium lead iodide. *Adv. Mater. Interfaces* **6**, 1801173 (2019).
2. Taylor, M., Urquhart, A.J., Zelzer, M., Davies, M.C. & Alexander, M.R. Picoliter Water contact angle measurement on polymers. *Langmuir* **23**, 6875-6878 (2007).
3. Xu, J. et al. Highly stretchable polymer semiconductor films through the nanoconfinement effect. *Science* **355**, 59-64 (2017).
4. Guo, Y.L., Yu, G. & Liu, Y.Q. Functional organic field-effect transistors. *Adv. Mater.* **22**, 4427-4447 (2010).
5. Xu, J. et al. Multi-scale ordering in highly stretchable polymer semiconducting films. *Nat. Mater.*, **18**, 594-602 (2019).
6. Oh, J.Y. et al. Intrinsically stretchable and healable semiconducting polymer for organic transistors. *Nature* **539**, 411-415 (2016).
7. Zheng, Y. et al. A molecular design approach towards elastic and multifunctional polymer electronics. *Nat. Commun.* **12**, 5701 (2021).
8. Zheng, Y. et al. An Intrinsically Stretchable high-performance polymer semiconductor with low crystallinity. *Adv. Funct. Mater.* **29**, 1905340 (2019).
9. Zhu, C., Wu, H.C., Nyikayaramba, G., Bao, Z. & Murmann, B. Intrinsically stretchable temperature sensor based on organic thin-film transistors. *IEEE Electron Device Lett.* **40**, 1630-1633 (2019).
10. Rao, Y.L. et al. Stretchable self-healing polymeric dielectrics cross-linked through metal-ligand coordination. *JACS* **138**, 6020-6027 (2016).
11. Shih, C.C., Lee, W.Y., Lu, C., Wu, H.C. & Chen, W.C. Enhancing the mechanical durability of an organic field effect transistor through a fluoroelastomer substrate with a crosslinking-induced self-wrinkled structure. *Adv. Electron. Mater.* **3**, 8 (2017).
12. Kang, B. et al. Stretchable polymer gate dielectric with segmented elastomeric network for organic soft electronics. *Chem. Mater.* **30**, 6353-6360 (2018).
13. Liu, K. et al. Low-Voltage Intrinsically Stretchable Organic Transistor Amplifiers for Ultrasensitive Electrophysiological Signal Detection. *Adv. Mater.* **35**, 2207006 (2022).
14. Liu, K. et al. Carbon nanotube-based van der Waals heterojunction electrodes for high-performance intrinsically stretchable organic photoelectric transistors. *Giant* **7**, 100060 (2021).
15. Bian, Y. et al. Spatially nanoconfined N-type polymer semiconductors for stretchable ultrasensitive X-ray detection. *Nat. Commun.* **13**, 7163 (2022).
16. Wang, W. et al. Strain-insensitive intrinsically stretchable transistors and circuits. *Nat. Electron.* **4**, 143-150 (2021).
17. Wang, S.H. et al. Skin electronics from scalable fabrication of an intrinsically stretchable transistor array. *Nature* **555**, 83-88 (2018).
18. Chen, M., Wang, C. & Hu, W. Organic photoelectric materials for X-ray and gamma ray detection: mechanism, material preparation and application. *J. Mater. Chem. C* **9**, 4709-4729 (2021).
19. Ciavatti, A. et al. Boosting direct X-Ray detection in organic thin films by small molecules tailoring. *Adv. Funct. Mater.* **29**, 1806119 (2018).
20. Gao, Y. et al. Ultrathin and ultrasensitive direct X-ray detector based on heterojunction phototransistors. *Adv. Mater.* **33**, e2101717 (2021).
21. Temiño, I. et al. Morphology and mobility as tools to control and unprecedentedly enhance X-ray sensitivity in organic thin-films. *Nat. Commun.* **11**, 2136 (2020).
22. Fraboni, B. et al. Organic semiconducting single crystals as next generation of low-cost, room-temperature electrical X-ray detectors. *Adv. Mater.* **24**, 2289-2293 (2012).
23. Wei, H. et al. Dopant compensation in alloyed $CH_3NH_3PbBr_{3-x}Cl_x$ perovskite single crystals for gamma-ray spectroscopy. *Nat. Mater.* **16**, 826-833 (2017).

24. Pan, W. et al. Hot-pressed $CsPbBr_3$ quasi-monocrystalline film for sensitive direct X-ray detection. *Adv. Mater.* **31**, 1904405 (2019).
25. Wei, W. et al. Monolithic integration of hybrid perovskite single crystals with heterogenous substrate for highly sensitive X-ray imaging. *Nat. Photonics* **11**, 315-321 (2017).
26. Deumel, S. et al. High-sensitivity high-resolution X-ray imaging with soft-sintered metal halide perovskites. *Nat. Electron.* **4**, 681-688 (2021).
27. Yakunin, S. et al. Detection of gamma photons using solution-grown single crystals of hybrid lead halide perovskites. *Nat. Photonics* **10**, 585-589 (2016).
28. Zhuang, R. et al. Highly sensitive X-ray detector made of layered perovskite-like $(NH_4)_3Bi_2I_9$ single crystal with anisotropic response. *Nat. Photonics* **13**, 602-608 (2019).
29. Pan, W. et al. $Cs_2AgBiBr_6$ single-crystal X-ray detectors with a low detection limit. Nat. Photonics 11, 726-732 (2017).
30. Thirimanne, H.M. et al. High sensitivity organic inorganic hybrid X-ray detectors with direct transduction and broadband response. *Nat. Commun.* **9**, 2926 (2018).
31. Mills, C.A. et al. Enhanced x-ray detection sensitivity in semiconducting polymer diodes containing metallic nanoparticles. *J. Phys. D.* **46**, 275102 (2013).
32. Ankah, G.N. et al. PbS quantum dot based hybrid-organic photodetectors for X-ray sensing. *Org. Electron.* **33**, 201-206 (2016).
33. Büchele, P. et al. X-ray imaging with scintillator-sensitized hybrid organic photodetectors. *Nat. Photonics* **9**, 843-848 (2015).
34. Xiang, L. et al. X-ray Sensitive hybrid organic photodetectors with embedded $CsPbBr_3$ perovskite quantum dots. *Org. Electron.* **98**, 106306 (2021).
35. Boroumand, F.A. et al. Direct x-ray detection with conjugated polymer devices. *Appl. Phys. Lett.* **91**, 033509 (2007).
36. Valitova, I., Hupman, A., Hill, I.G. & Syme, A. Poly(3-hexylthiophene-2,5-diyl) based diodes for ionizing radiation dosimetry applications. *Org. Electron.* **88**, 105981 (2021).
37. Basiricò, L. et al. Direct X-ray photoconversion in flexible organic thin film devices operated below 1 V. *Nat. Commun.* **7**, 13063 (2016).
38. Yi, L., Hou, B., Zhao, H., Tan, H.Q. & Liu, X. A double-tapered fibre array for pixel-dense gamma-ray imaging. *Nat. Photonics* **17**, 494–500 (2023).
