# Supplementary material for: A detachable interface for stable low-voltage stretchable transistor arrays and high-resolution X-ray imaging
Source: Nat Commun. 2024 Mar 23;15:2624. doi: 10.1038/s41467-024-47026-9 (PMC10960804; doi:10.1038/s41467-024-47026-9)
Supplement: Supplementary file 3 — Description of Additional Supplementary Files [file 41467_2024_47026_MOESM3_ESM.pdf]

Description of Additional Supplementary Files

Supplementary Movie 1: Simulation of the pristine transfer process.
Supplementary Movie 2: Simulation of the LiF-assisted transfer process.
